# Electroporation as a strategy to improve the efficacy of chemotherapy in neuroblastoma: an in vitro study

**DOI:** 10.2478/raon-2026-0028

**Published:** 2026-06-26

**Authors:** Jonathan J Neville, Laura Privitera, Jingchen Sun, Renata Da Costa Magueta, Piotr Golda, Adam C Sedgwick, Paolo De Coppi, Ismael Diez Perez, Premal A Patel, John Anderson, Stefano Giuliani

**Affiliations:** 1 University College London, Great Ormond Street Institute of Child Health, London, UK; 2 Department of Specialist Neonatal and Paediatric Surgery, Great Ormond Street Hospital, London, UK; 3 Division of Biosciences, Faculty of Life Sciences, University College London, London, UK; 4 London Metallomics Facility, King’s College London, London, UK; 5 Department of Chemistry, King’s College London, London, UK; 6 Interventional Radiology, Radiology Department, Great Ormond Street Hospital, London, UK; 7 Department of Paediatric Oncology, Great Ormond Street Hospital, London, UK

**Keywords:** paediatrics, oncology, electroporation, cisplatin, neuroblastoma, poly-(ADP ribose) polymerase 1

## Abstract

**Background:**

Reversible electroporation involves the use of pulsed electric fields to temporarily disrupt and permeabilise cell membranes. Electroporation combined with chemotherapy - electrochemotherapy (ECT) - increases tumour cell permeability to chemotherapeutic drugs and enhances their effect. We investigated the efficacy of electroporation and cisplatin as a treatment for neuroblastoma *in vitro*, and explored whether inhibition of DNA repair mechanisms with olaparib potentiates its effects.

**Materials and methods:**

Three immortalised neuroblastoma cell lines were exposed to eight 1 ms square wave pulses of 0–0.93 kV/cm electric fields in the presence of propidium iodide and reversible electroporation was identified by detecting live propidium iodide positive cells by flow cytometry. Intracellular cisplatin and olaparib levels after electroporation were investigated by measuring intracellular platinum via inductively coupled plasma mass spectrometry and by quantifying a fluorescent olaparib with flow cytometry. Cells were exposed to electroporation in the presence of cisplatin and olaparib, and cell death was quantified by flow cytometry. Presence of DNA damage was evaluated by quantifying γ-H2AX immunofluorescence.

**Results:**

Reversible electroporation (0.74 kV/cm) increased intracellular cisplatin and olaparib levels. Electroporation with cisplatin (1–100 μM) significantly reduced cell viability compared to cisplatin treatment in all cell lines. The addition of olaparib (5 μM) modestly potentiated the effects of cisplatin and electroporation. DNA damage was significantly higher following treatment with a combination of electroporation, cisplatin and olaparib, compared to each treatment alone.

**Conclusions:**

Electroporation with cisplatin appears effective against neuroblastoma *in vitro*. Further work will investigate the efficacy of electroporation with cisplatin using clinical ECT devices.

## Introduction

Neuroblastoma is the most common extracranial solid tumour in children and is responsible for 10–15% of all paediatric cancer mortality.^1^ High-risk disease associates with poor outcomes and current 5-year survival rates are 50%.^2^ The treatment of neuroblastoma is multi-modal, and includes chemotherapy, radiotherapy, surgery and immunotherapy.^3^ Chemotherapy and radiotherapy are both associated with significant long-term morbidity in children.^4^ Chemotherapy is associated with the development of immunological, cardiovascular, renal, pulmonary, and haematological morbidities, while radiotherapy increases the risk of children developing a second malignancy in a dose-dependent manner. In inoperable high-risk neuroblastoma, with image-defined risk factors, there is a need for targeted therapies that can downstage the tumour whilst minimising potential side effects and long-term sequelae. Incomplete surgical resection occurs in approximately 20–30% of high-risk neuroblastoma cases, and associates with an increased cumulative incidence of local progression, and reduced event-free and overall survival.^3,5^ Therefore, the introduction of image-guided and intra-operative treatments that can target residual tumour are necessary.

Electroporation of cells is performed using intense short duration pulses of electricity to disrupt the cell plasma membrane and enhance its permeability.^6^ Permanent changes to the cell membrane which cause cell death, termed irreversible electroporation, occur with higher field strengths and longer applications of electricity. In contrast, applications of lower strength electric fields result in reversible electroporation. Here, cells recover after a temporary period of increased permeability, during which molecules are able to enter the cell that would otherwise be unable to. In a clinical context, electrochemotherapy (ECT) combines reversible electroporation of tumour cells with either localised or systemic chemotherapy to increase the uptake of poorly cell-permeable cytotoxic drugs.^7,8^ This improves the efficacy of the chemotherapy whilst limiting systemic side effects and reducing long-term morbidity.

ECT is a well-established treatment in paediatrics and oncology. Current clinical indications for ECT in children include the treatment of benign and malignant tumours, as well as vascular and lymphatic malformations.^9^-^13^ Multiple chemotherapy agents are used, including bleomycin and cisplatin. There is also emerging pre-clinical evidence suggesting that the addition of poly-(ADP ribose) polymerase (PARP) inhibitors, such as olaparib, may enhance the effects of ECT.^14^ PARP is a ubiquitous nuclear protein involved in the detection and repair of DNA damage. *MYCN*-amplified neuroblastoma cells show an intrinsic therapeutic vulnerability to PARP inhibition characterised by heightened replication stress and significant upregulation of PARP.^15,16^ PARP inhibition may augment the effects of ECT in *MYCN* -amplified neuroblastoma by preventing the repair of ECT-induced DNA damage.

In inoperable neuroblastoma, ECT may have a role in management by decreasing tumour volume and reducing vascularity, which may improve symptoms or downstage image-defined risk factors for surgery. In addition, it may be useful to treat residual neuroblastoma that remains after maximal safe resection intra-operatively. To date there are limited studies investigating ECT for the treatment of neuroblastoma.^17^ We aimed to investigate the efficacy of electroporation combined with cisplatin as a potential treatment for neuroblastoma and explore whether the effects were potentiated by PARP1 inhibition.

## Materials and methods

### Cell lines and culture conditions

Immortalised human neuroblastoma cell lines with *MYCN*-amplification (KELLY, LAN-1 and SK-N-BE), kindly provided by Professor John Anderson (University College London), were cultured in Roswell Park Memorial Institute (RPMI) 1640 medium (Merck Life Science) supplemented with 10% foetal bovine serum (FBS; Thermo Fisher Scientific) and 1 % GlutaMAX (Gibco). Cells were detached using trypsin-ethylenediaminetetraacetic acid (EDTA) solution (Gibco) for passaging at 70–80% confluence every 3–4 days or immediately prior to electroporation. Cells lines were incubated in a humidified incubator at 37°C and 5% carbon dioxide. Cells were used up to a passage number of 30 and tested regularly for mycoplasma.

### Electroporation

**FIGURE 1.**
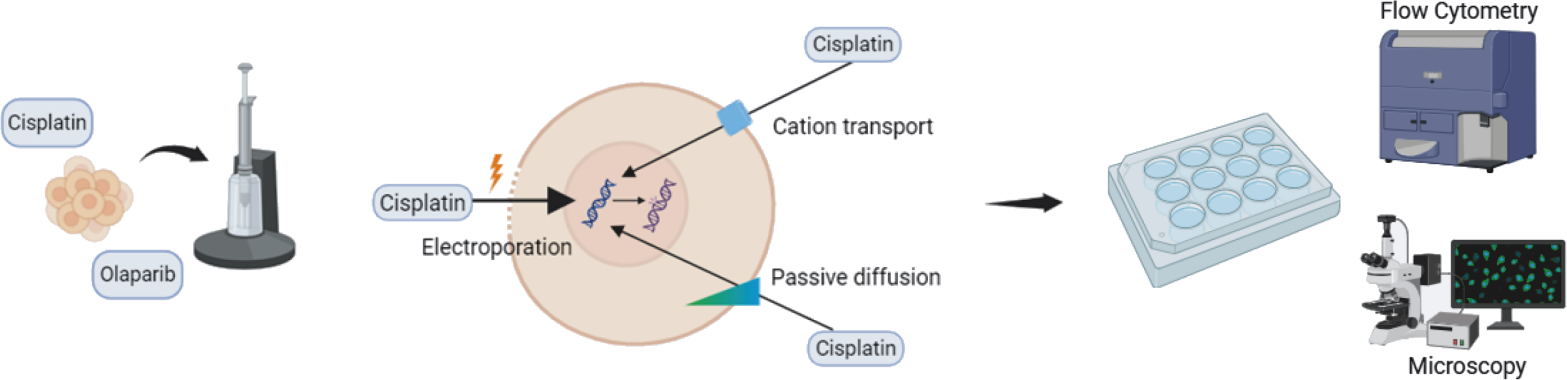
Overview of the study design. Electroporation was modelled using the Neon Transfection system (left). Cell suspensions were electroporated in the presence of cisplatin and olaparib treatment. The mechanism of electrochemotherapy (centre): electroporation increases intracellular cisplatin levels by reversibly permeabilising the cell plasma membrane. Following treatment, cells were assayed using flow cytometry and immunofluorescence microscopy (right). Figure was created in BioRender (www.biorender.com).

Electroporation was performed using the Invitrogen Neon Transfection System (Figure 1). Single cell suspensions were electroporated in a uniform electric field (0–2.5 kV) as per manufacturer’s instructions. In short, single cell suspensions of 1x10^5^ cells in 100 μL electroporation buffer were loaded into the Neon pipette. The Neon pipette was loaded into the pipette station, and eight 1 ms square wave pulses of a 0.19–0.93 kV/cm electric field were applied to the cell suspension. Following electroporation, cells were immediately placed into pre-warmed medium and cultured for up to 48 hours post-electroporation prior to analysis. Drugs were administered at the time of electroporation.

### Flow cytometry

Following electroporation, cells were detached using Trypsin-EDTA and resuspended in phosphate buffered saline (PBS) supplemented with 1% FBS and 0.1 mM EDTA. To investigate the efficacy of electroporation at enhancing cell permeabilisation and identify the optimal experimental conditions for further toxicity studies, cells were electroporated in the presence of 10 μL of 10 μg/ml propidium iodide (PI). PI is a cell membrane impermeable dye in standard conditions. Cells were counterstained with 0.5 μg/ml 4’,6-diamidino-2-phenylindole (DAPI) to identify live cells.

Similarly, to confirm the increased uptake of olaparib following electroporation, a previously described fluorescently labelled olaparib (PARPi-FL) was used (Tocris Bioscience).^18^ Cells were electroporated in the presence of 5 μM PARPi-FL and uptake was assessed by comparing these cells to a control group treated with 5 μM PARPi-FL without electroporation. The dose was selected based on published literature.^14–16^ Cells were counterstained with 0.5 μg/ml DAPI to identify live cells. Data acquisition was performed on a FACSymphony A5 flow cytometer (BD Biosciences). Analysis was performed using FlowJo software (v10.10.0, TreeStar).

### Mass spectrometry

To determine whether electroporation results in an increased uptake of cisplatin, KELLY and LAN-1 neuroblastoma cells were treated with cisplatin and electroporation (10 μM cisplatin, 0.74 kV/cm) and intracellular platinum was subsequently measured using inductively coupled plasma triple quadrupole mass spectrometry (ICP-MS).

Briefly, following electroporation and cisplatin treatment cells were allowed to recover for four hours in pre-warmed RPMI. Cells were counted using a Countess 3 cell counter (Invitrogen), pelleted by centrifugation at 400 rcf for 5 minutes and digested with concentrated nitric acid (65–70% w/w, trace metal grade, Fisher Chemical) at 60°C for two hours. After digestion, samples were spiked with an iridium (^193^Ir) stock solution as internal standard (High Purity Standards). Samples were diluted to a final volume of 1.48 mL with ultrapure MilliQ water (18.2 MW) to achieve a final concentration of 0.1 M nitric acid and 10 ppb ^193^Ir.

All measurements were conducted on a Perkin Elmer NexION 5000 ICP-MS under Dynamic Reaction Cell mode at the London Metallomics Facility, King’s College London. The introduction system to the instrument was a Cetac ASX-560 autosampler coupled to a SeaSpray glass nebuliser, fitted to a quartz cyclonic spray chamber. Argon plasma flow and nebuliser gas flow rates were 18 L/min and 0.98 L/min, respectively. A calibration curve was prepared by serial dilutions of an ICP-MS Platinum standard (High Purity Standards) in a range of 0.05–500 ppb of platinum (^195^Pt) and measurements were taken in Kinetic Energy Discrimination mode with a 0.5 mL/min flow of helium inside the reaction cell.

Quality control of ICP-MS measurements was ensured through a combination of repeat measurements of acid blanks and a calibration standard, repeated at intervals during the analysis. Analyte measurements were normalised to the internal standard ^193^Ir to account for instrument drift and matrix effects, and subsequently blank corrected by subtracting the average analyte intensity of repeat blank samples. The corrected isotopic intensity was converted to concentration measurements by regression analysis using the calibration curve. The quality of the regression analysis was confirmed by verifying the linearity of the calibration curve. The final concentration of platinum in the cells was expressed in μg ^195^Pt per gram normalised to the number of live cells.

### Fluorescence microscopy

γ-H2AX foci immunofluorescence staining was performed to identify and quantify DNA damage in neuroblastoma cell lines 48-hours following electroporation and treatment with cisplatin and/or olaparib.^19^ Immediately post-treatment 1x106 cells were seeded onto 23 mm glass-bottom cell culture dishes (Thermo Fisher Scientific) containing pre-warmed RPMI and treatment doses of cisplatin and/or olaparib. Forty-eight hours posttreatment, cells were stained for γ-H2AX foci. Cells were washed with PBS and fixed with 4% paraformaldehyde (PFA) for 30 minutes at room temperature. After PFA quenching with ammonium chloride solution, cells were incubated in blocking solution (1% bovine serum albumin and 0.1% Triton in PBS) for one hour and then incubated with γ-H2AX (S139) antibody (1:100; 3F2; Thermo Fisher Scientific, #MA1-2022) overnight at 4°C. Cells were then washed with 0.1% Triton in PBS and incubated with AlexaFluor488 secondary antibody (1:200; Thermo Fisher Scientific, A11006) for four hours at room temperature. Nuclei were counterstained with Hoechst 33342 (1:500). Cells were imaged on a Nikon Eclipse Ti2 microscope.

Quantification of γ-H2AX foci fluorescence was performed using Fiji software.^20^ Cell nuclei identification and segmentation were performed using the DAPI channel. γ-H2AX foci were then localised within the segmented nuclei and quantified. Thresholds for γ-H2AX foci signal was set using Max Entropy at 1%. The number of γ-H2AX foci per nucleus was measured and averaged across six separate images for each experimental condition.

### Statistical analysis

Continuous variables were compared using two-tailed unpaired t-tests or two-way ANOVA. Analysis was performed in GraphPad Prism (v10, GraphPad Software, San Diego, USA). Information and data are presented according to recommendations on best practice for reporting applications of electric pulse delivery to biological tissues.^21^ All experiments were performed in triplicate.

## Results

### Reversible electroporation of neuroblastoma cell lines

**FIGURE 2.**
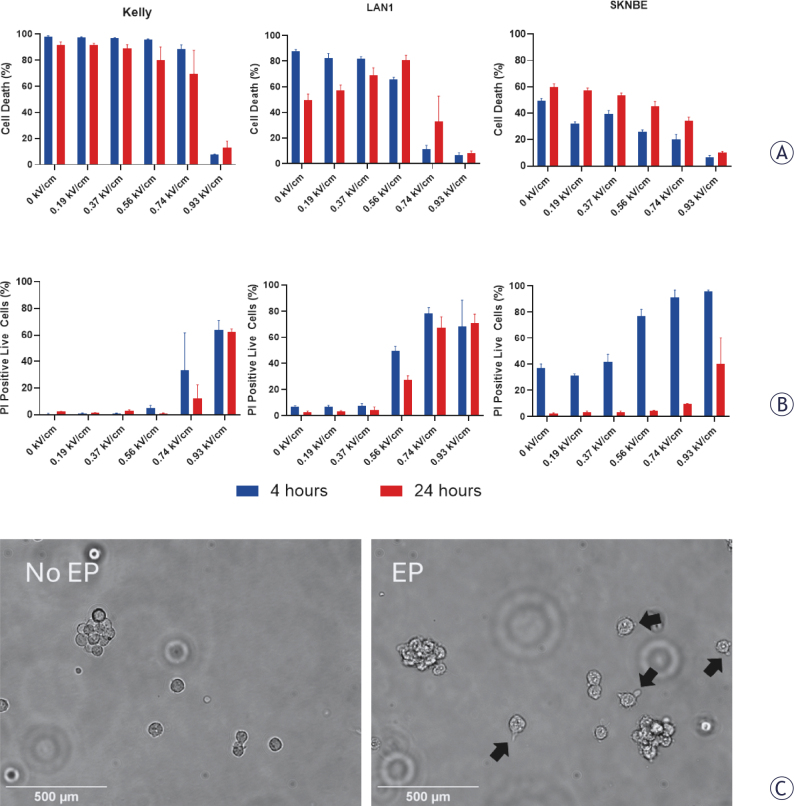
Electroporation (EP) increases neuroblastoma cell permeability. **(A)** Across three neuroblastoma cell lines (KELLY, LAN-1 and SK-N-BE) electroporation at increasing electric field strengths (0 - 0.93 kV/cm) increased cell death at immediate (4 hour, blue) and 24-hour (red) timepoints. **(B)** Increasing electric field strengths (0 - 0.93 kV/cm) increased cell permeability to propidium iodide (PI). **(C)** Representative images of LAN-1 cells in suspension before and after electroporation (0.93 kV/cm) showing blebs forming on the cell membrane (black arrows). Data are presented as mean ± standard deviation. Scale bars 500 μm.

Permeabilisation of neuroblastoma cell lines by electroporation at increasing voltages (0 kV/cm, 0.19 kV/cm, 0.37 kV/cm, 0.56 kV/cm, 0.74 kV/cm and 0.93 kV/cm) was determined by identifying live neuroblastoma cells positive for PI on flow cytometry (Figure 2A and B). Irreversible electroporation, defined as > 90% cell death at 4 hours, was observed at 0.93 kV/cm in all cell lines. Electroporation was reversible at 0.74 kV/cm in KELLY cells but still caused death in > 50% cells in LAN-1 and SK-N-BE cells at 4 hour and 24-hour time points. Effective permeabilisation was achieved at 0.74 kV/cm in KELLY and SK-N-BE cells, and 0.56 kV/cm in LAN-1 cells. To maintain a consistent treatment across cell lines, 0.74 kV/cm was chosen for further experiments to balance cell viability with sufficient cell permeabilisation. Brightfield imaging of treated cells confirmed cell membrane disruption post-electroporation, with the formation of cell surface blebs (Figure 2C).

**FIGURE 3.**
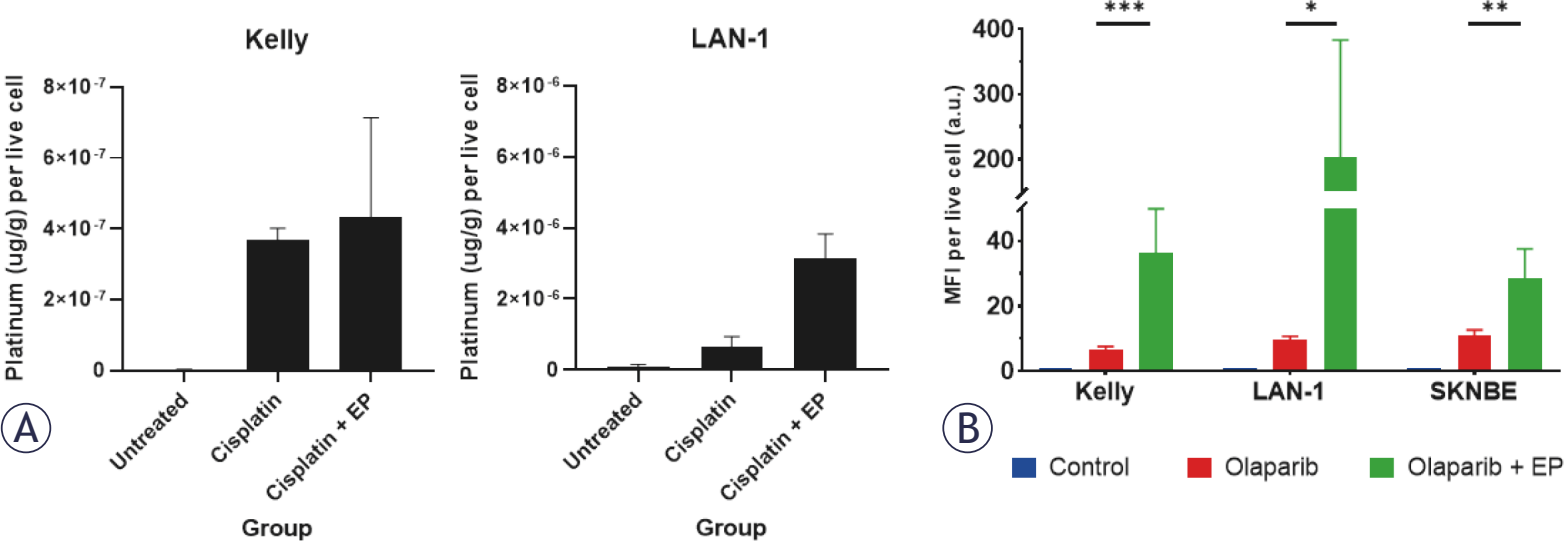
**(A)** Electroporation (EP, 0.74 kV/cm) with cisplatin (10 μM) increases intracellular platinum content (μg/g) versus treatment with cisplatin alone in KELLY and LAN-1 cells. Untreated cells received no cisplatin. **(B)** Electroporation (0.74 kV/cm) with a fluorescent-labelled olaparib (5 μM) compared to treatment with the fluorescent-labelled olaparib alone significantly increases fluorescence intensity per live cell in KELLY, LAN-1 and SK-N-BE cells. Data are presented as mean ± standard deviation. Statistical comparison by one-way ANOVA with Tukey’s post-hoc test. * p <0.05; ** p <0.01; *** p <0.001.

To assess intracellular cisplatin uptake following electroporation cells were treated with cisplatin (10 μM) with and without electroporation (0.74 kV/cm). Platinum level per number of live cells was then quantified by ICP-MS. In both KELLY and LAN-1 cells, increased intracellular cisplatin accumulation following electroporation was observed (Figure 3A).

Similarly, increased intracellular olaparib accumulation following electroporation (0.74 kV/cm) was inferred by detecting PARPi-FL fluorescence in cells via flow cytometry. The mean fluorescence intensity of PARPi-FL normalised to cell number was significantly higher following electroporation compared to drug treatment alone in KELLY (36.3 *vs*. 6.6, p < 0.0001), LAN-1 (202.3 *vs*. 9.8, p < 0.05) and SK-N-BE (28.4 *vs*. 11.0, p < 0.01) cells (Figure 3B), suggesting increased intracellular olaparib following electroporation.

### Toxicity of electroporation combined with cisplatin in neuroblastoma cell lines with and without PARP1 inhibition

**FIGURE 4.**
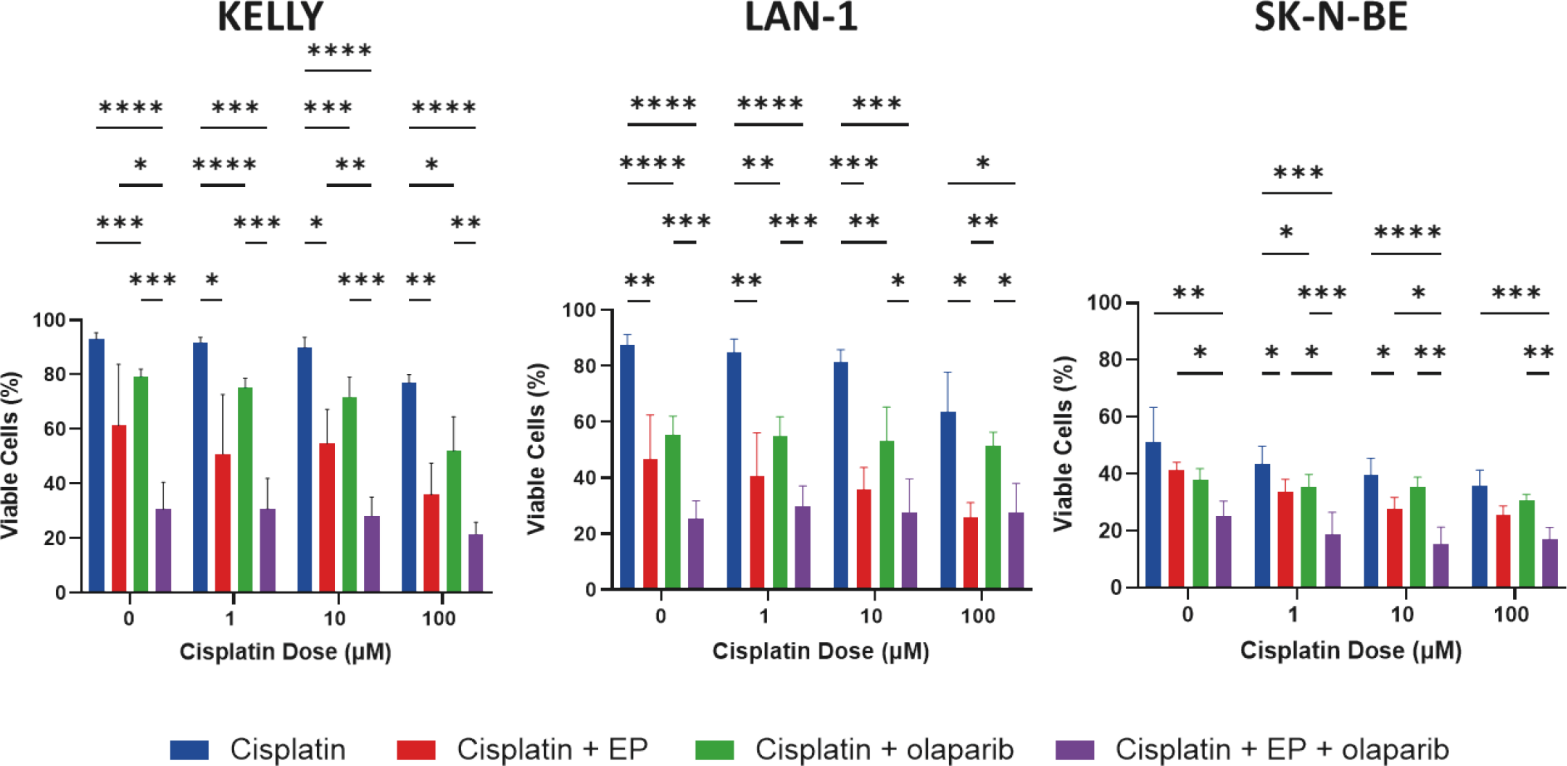
Electroporation (EP, 0.74 kV/cm) with cisplatin is significantly more effective than cisplatin alone at killing neuroblastoma cell lines (KELLY, LAN-1 and SK-N-BE). The addition of olaparib increases toxicity. Data are presented as mean ± standard deviation. Statistical comparison by one-way ANOVA with Tukey’s post-hoc test. *p <0.05; **p <0.01; *** p <0.001; **** p <0.0001.

Neuroblastoma cells were assessed via flow cytometry 48 hours after treatment with increasing doses of cisplatin (0 μM, 1 μM, 10 μM and 100 μM), with and without electroporation (0.74 kV/cm). Compared to cisplatin alone, electroporation and cisplatin significantly reduced the proportion of viable neuroblastoma cells in all three cell lines at 48 hours (Figure 4). The combination of electroporation and cisplatin plus PARP1 inhibition with olaparib (5 μM) further reduced cell viability across all three cell lines compared to cisplatin alone. However, the additional toxicity compared to electroporation and cisplatin was weakly additive at 48 hours.

### PARP1 inhibition prevents the repair of cisplatin-induced DNA damage

**FIGURE 5.**
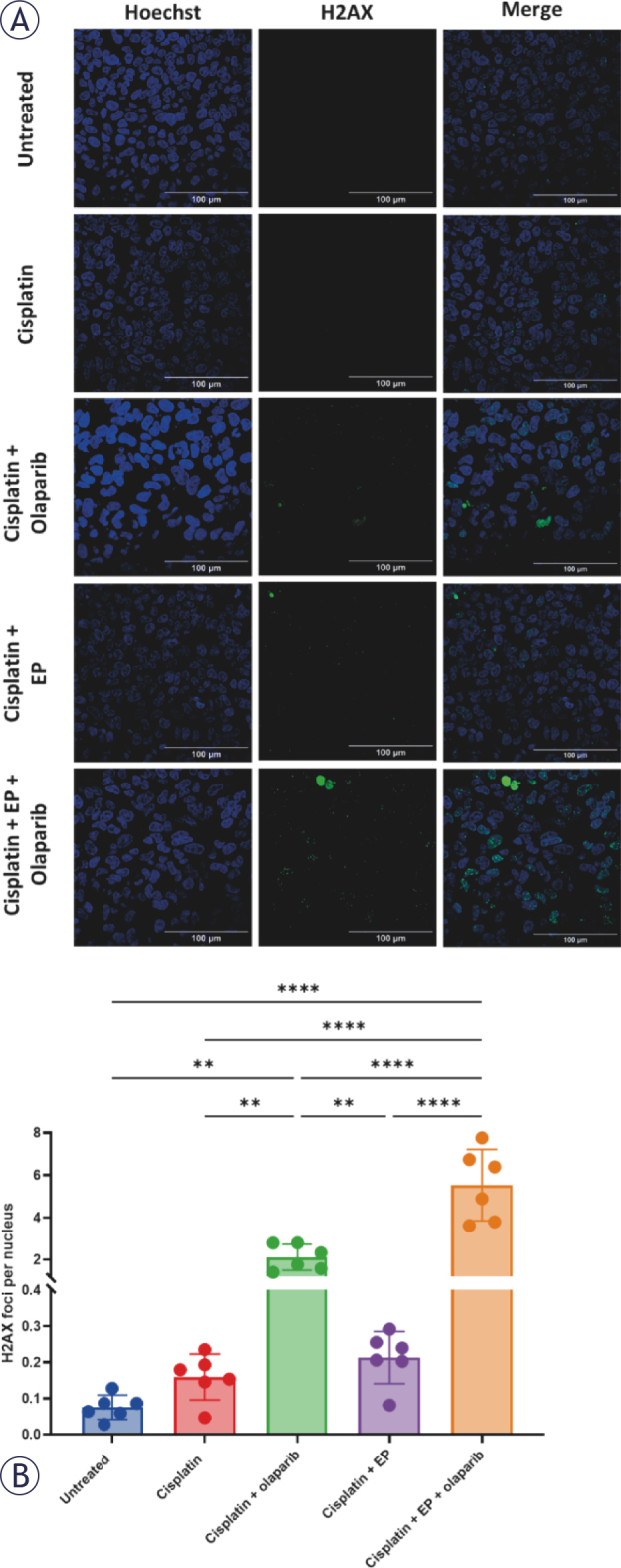
**(A)** Representative γ-H2AX foci staining 48 hours following treatment with electroporation (EP, 0.74 kV/cm) and cisplatin (10 μM) with and without olaparib (5 μM) in KELLY cells. **(B)** Olaparib significantly increases the number of γ-H2AX foci per nucleus. Data are presented as mean ± standard deviation. Statistical comparison by one-way ANOVA with Tukey’s post-hoc test. *p <0.05; **p <0.01; *** p <0.001; **** p <0.0001

To evaluate DNA damage, γ-H2AX foci staining was performed at 48-hours post-treatment (Figure 5A). The mean number of γ-H2AX foci per nucleus was significantly higher in the ECT (0.74 kV/cm, 10 μM cisplatin) and olaparib (5 μM) group compared to cisplatin alone (5.5 *vs*. 0.16, p < 0.001) and electroporation and cisplatin (5.5 *vs*. 0.21, p < 0.001; Figure 5B). Notably, cisplatin (10 μM) with olaparib (5 μM) also increased the number of γ-H2AX foci per nucleus compared to cisplatin alone (2.1 *vs*. 0.16, p < 0.001). These results suggest that electroporation increases cisplatin-induced DNA damage, and that inhibition of DNA damage repair with olaparib enhances this effect.

## Discussion

ECT is a well-established treatment in interventional radiology, however there is a paucity of pre-clinical and clinical evidence regarding its potential application to neuroblastoma. This study demonstrates that electroporation with cisplatin is an effective treatment against neuroblastoma *in vitro*. We have shown that electroporation of neuroblastoma cell lines results in increased intracellular cisplatin and olaparib. Electroporation with cisplatin significantly enhances neuroblastoma cell death compared to cisplatin alone. Furthermore, our results demonstrate that the combination of electroporation and cisplatin with PARP1 inhibition exerts a small additive effect through the inhibition of cisplatin-induced DNA repair.

Few studies have investigated the effects of combining electroporation with chemotherapy on paediatric solid tumours. Our findings corroborate a previous study that reported that cisplatin paired with electroporation shows efficacy at killing neuroblastoma cell lines. Esmekaya *et al*. observed significantly greater efficacy of cisplatin ECT (0.15–24 μg/mL cisplatin, eight 100 μs pulses of 1.5 kV/cm) compared to cisplatin alone in a single neuroblastoma cell line, SH-SY5Y (17). However, they did not confirm increased cisplatin uptake nor investigate the mechanism of action.

Isobe and colleagues combined electroporation with methotrexate to treat osteosarcoma in an allografted subcutaneous murine model (22). Mice treated with ECT (10 mg/kg intraperitoneal methotrexate, eight 75 ms pulses of 0.1 V/cm) showed reduced tumour growth compared to no treatment, methotrexate or electroporation alone. Four mice (57%) in the ECT group achieved a complete response. The authors observed a two-fold increase in the intra-tumoral methotrexate concentration in the ECT group compared to methotrexate alone.

The combination of electroporation and chemotherapy with PARP1 inhibition has been investigated in human breast cancer cell lines. Bosnjak and colleagues tested electroporation with cisplatin and bleomycin *in vitro* in a *BRCA* negative oestrogen receptor positive breast cancer cell line and observed that olaparib increased efficacy of bleomycin and electroporation but not cisplatin.^23^ Further work by the same group then identified that a *BRCA* mutated breast cancer cell line was sensitive to combination therapy of electroporation and cisplatin paired with olaparib. However, the relationship was shown to be additive, not synergistic, suggesting that olaparib did not augment the effects of treatment.^14^ Further, in a second non-*BRCA* mutated breast cancer line, no increased toxicity with olaparib in combination with electroporation and cisplatin or bleomycin, was observed. Similarly, in this study we have shown that electroporation with cisplatin and olaparib is marginally more effective at killing neuroblastoma cells than electroporation and cisplatin alone. Tumours with defective DNA repair mechanisms are more susceptible to PARP1 inhibition. Despite *MYCN*-amplified neuroblastoma showing sensitivity to PARP inhibition, the lack of dysfunctional DNA repair mechanisms in neuroblastoma cell lines may explain the modest additive effect observed. Further work is required to understand why there may be a variable response to olaparib across cell types with intact DNA repair mechanisms. Similarly, clinical trials assessing the safety of a combination of ECT, cisplatin and olaparib in patients would be required prior to widespread adoption.

Multiple reports of clinical electroporation with chemotherapy (ECT) in children have been published, highlighting its safety and efficacy for the treatment of benign and malignant disease. ECT has been applied to malignant skin lesions in children, including squamous and basal cell carcinoma of the head and neck, showing a complete clinical response.^10,11^ ECT has also been used in children to reduce the volume of venous and lymphatic malformations.^9,12,24,25^ Given the established safety profile of ECT in paediatric patients, further work should focus on investigating the safety and clinical efficacy of ECT in children for treating inoperable and surgical residual neuroblastoma. Clinical translation will require thorough assessment of optimal ECT parameters, including field strength, electrode array type and distribution, combined with prospective reporting of procedural and oncological outcomes, and complications.

This work is limited by its use of immortalised two-dimensional neuroblastoma cell lines and *in vitro* setting. As such, we have not investigated the efficacy of electroporation and cisplatin, nor ECT, in the context of the complete tumour microenvironment or in a model incorporating the immune system. The optimised electrical parameters used in this study are specific to the model system used. For *in vivo* translation, optimisation of electric fields in neuroblastoma tissue will be required. In addition, we have only investigated the efficacy of cisplatin. Other chemotherapy drugs are used during clinical ECT, such as bleomycin. Further work should expand investigations to include other chemotherapeutics and combination therapies.

In conclusion, electroporation combined with cisplatin shows promise as a treatment for neuroblastoma. ECT may be useful as an adjunctive treatment for inoperable neuroblastoma or surgical residual disease. Further work should investigate the safety and efficacy of ECT in children with inoperable neuroblastoma in a phase one clinical trial. Combination therapy of ECT with olaparib should also be considered.
